# Bone Density and Trabecular Bone Score Decline Rapidly in the First Year After Bone Marrow Transplantation with a Marked Increase in 10-Year Fracture Risk

**DOI:** 10.1007/s00223-024-01189-1

**Published:** 2024-02-20

**Authors:** Joanna Y. Gong, Cherie Chiang, John D. Wark, David Ritchie, Yvonne Panek-Hudson, Minh V. Le, Lydia Limbri, Nicolo Fabila, Spiros Fourlanos, Christopher J. Yates

**Affiliations:** 1 Department of Diabetes & Endocrinology, Royal Melbourne Hospital, 300 Grattan Street, Parkville, Melbourne, VIC 3052 Australia; 2 Department of Endocrinology & Diabetes, Western Health, Melbourne, Australia; 3 Endocrinology Department, Austin Health, Melbourne, Australia; 4 Department of Medicine (RMH), University of Melbourne, Melbourne, Australia; 5 Clinical Haematology, Peter MacCallum Cancer Centre and Royal Melbourne Hospital, Melbourne, Australia; 6 Northeast Health Wangaratta, Victoria, Australia

**Keywords:** Bone density, Osteoporosis, Fracture, Post-transplant care, Trabecular bone score

## Abstract

As outcomes from allogeneic bone marrow transplantation (BMT) have improved, prevention of long-term complications, such as fragility fractures, has gained importance. We aimed to assess areal bone mineral density (aBMD) and trabecular bone score (TBS) changes post BMT, and determine their relationship with fracture prevalence. Patients who attended the Royal Melbourne Hospital (RMH) BMT clinic between 2005–2021 were included. Patient characteristics and dual-energy X-ray absorptiometry (DXA) values were collected from the electronic medical record and a survey. TBS iNsight™ was used to calculate TBS for DXA scans performed from 2019 onwards. 337 patients with sequential DXAs were eligible for inclusion. Patients were primarily male (60%) and mean age ± SD was 45.7 ± 13.4 years. The annualised decline in aBMD was greater at the femoral neck (0.066g/cm^2^ (0.0038–0.17)) and total hip (0.094g/cm^2^ (0.013–0.19)), compared to the lumbar spine (0.049g/cm^2^ (− 0.0032–0.16)), *p* < 0.0001. TBS declined independently of aBMD T-scores at all sites. Eighteen patients (5.3%) sustained 19 fractures over 3884 person-years of follow-up post-transplant (median follow-up 11 years (8.2–15)). This 5.3% fracture prevalence over the median 11-year follow-up period is higher than what would be predicted with FRAX® estimates. Twenty-two patients (6.5%) received antiresorptive therapy, and 9 of 18 (50%) who fractured received or were on antiresorptive therapy. In BMT patients, aBMD and TBS decline rapidly and independently in the first year post BMT. However, FRAX® fracture probability estimates incorporating these values significantly underestimate fracture rates, and antiresorptive treatment rates remain relatively low.

**Supplementary Information:**

The online version contains supplementary material available at 10.1007/s00223-024-01189-1.

## Introduction

As outcomes from allogeneic bone marrow transplantation (BMT) continue to improve, monitoring for longer-term complications becomes increasingly important. Decline in bone health post BMT is well-established [1], although the pathophysiology is multifactorial. BMT preparative chemotherapy and total body irradiation, glucocorticoids (short-term or prolonged for graft-versus-host disease (GVHD)), suboptimal nutrition and hypogonadism may all contribute [2]. Increased rates of infection due to poor graft function have been associated with reduction in areal bone mineral density (aBMD) in some studies, while other studies have associated infection with greater aBMD [3, 4].

There are limited longitudinal data in the BMT population to guide bone health assessment and timing of antiresorptive therapy. Probability of fracture is high, but currently available fracture probability calculators only have modest predictive ability in these patients and do not include BMT as a risk factor [5].

Antiresorptive therapies are efficacious to prevent bone loss post BMT, but the frequency of surveillance dual energy X-ray absorptiometry (DXA) and the trigger for treatment initiation are still to be determined [6]. Additional determinants of fracture risk in BMT have not been explored. Trabecular bone score (TBS) is a novel tool used to assess bone microarchitecture, which is a key component of bone quality that is not accounted for by DXA-derived aBMD alone. It is a grey-level textural measurement that is derived from the DXA lumbar spine image and can assess fracture risk in chronic kidney disease metabolic bone disease [7].

We aimed to assess aBMD and TBS changes post BMT and to determine their relationship with probability of fracture, in order to establish optimal timing for DXA screening and facilitate appropriate intervention with antiresorptive therapy.

## Materials and Methods

We performed a retrospective cohort study of patients who underwent BMT at the Royal Melbourne Hospital (RMH) and had undergone sequential DXA scans pre and post BMT, or at least two DXA scans post BMT. Baseline, pre-BMT DXA readings were not available for some patients. In these cases, DXA scans pre-BMT were not performed or not available in the medical record. This was partly due to some transplants occurring as early as 1992, at the dawn of DXA in routine clinical practice. All patients with TBS data had a pre-BMT DXA scan available.

The cohort comprised two groups of patients; the first being all patients who attended the RMH BMT clinic from 2005–2018 and responded to an electronic or paper-based survey sent in 2022 (see Supplementary Appendix 1); the second consisted of BMT recipients who had a DXA scan performed after TBS software became available at RMH between 2019 − 2021. These two cohorts were combined to create the final patient population for analysis.

Patient characteristics including low-trauma fracture data and DXA values were collected in 2022 from the electronic medical record and surveys sent to consenting patients. TBS iNsight^TM^ (Medimaps SA, France) was used to calculate TBS, and FRAX® and FRAX®-TBS were used to calculate probability of fracture.

Data were synthesised and analysed, and graphs created using R version 4.2.2 (R Project for Statistical Computing). The Shapiro–Wilk test was used to assess normality, with the Wilcoxon test used to analyse non-normally distributed continuous variables and the t-test for normally-distributed continuous variables. The Chi-squared test was used to analyse categorical variables and the Friedman’s test was used to analyse non-parametric dependent measures. Logistic regression was employed for multivariate analyses performed to predict the probability of fracture based on predictor variables. All models were assessed to be well-fit as per the Hosmer–Lemeshow goodness-of-fit test and there was no collinearity present between predictive variables in any of the models. Normally-distributed data were expressed as mean ± standard deviation (SD) and non-normally distributed data were expressed as median (interquartile range (IQR)).

## Results

In total, 337 patients were eligible for inclusion, of whom 47 had sequential TBS available. Of the 337 patients, 245 patients (73%) had their first DXA scan completed prior to BMT. First DXA scans performed prior to BMT were performed a median (IQR) of 30 days (22–36) prior to transplant, and first DXA scans performed after BMT were performed a median (IQR) of 1.25 years (0.28–7.5) following BMT. Of the 287 patients sent a survey, 238 responded (83% response rate). Patients were followed for a median of 11 years (8.2–15) post BMT, totalling 3884 person-years of follow-up.

Patients had a mean age of 45.7 ± 13.4 years at the time of BMT, and they were primarily male (201/337, 59.6%). The median BMI was 25.8 kg/m^2^ (22.8–29.3). Characteristics of subjects who did or did not fracture are displayed in Table 1.

**Table 1:** Characteristics of 337 patients who underwent bone marrow transplant (BMT), separated by the presence or absence of osteoporotic fracture post BMT

	Fracture (*N* = 18)	No fracture (*N* = 319)	Total (*N* = 337)	*p*
Gender				
Male	6 (33.3)	195 (61.1)	201 (59.6)	0.036
Female	12 (66.7)	124 (38.9)	136 (40.4)	
Age (years) at BMT	51.9 ± 10.2	45.3 ± 13.5	45.7 ± 13.4	0.024
Weight (kg)^a^	75.0 (68.0–84.0)	76.0 (65.0–88.3)	76.0 (65.0–88.0)	0.83
Male	84.0 (75.0–84.0)	81.0 (70.0–90.0)	81.0 (70.1–90.0)	0.67
Female	70.0 (65.8–80.8)	67.0 (59.0–80.5)	67.0 (60.0–80.5)	0.39
Height (cm)^a^	171 ± 8.51	171 ± 10.0	171 ± 9.93	0.93
Male	180 ± 4.45	177 ± 7.54	177 ± 7.47	0.16
Female	166 ± 5.59	163 ± 7.51	164 ± 7.39	0.16
BMI (kg/m^2^)^a^	26.1 (22.6–29.3)	25.8 (22.8–29.3)	25.8 (22.8–29.3)	0.96
Time at first DXA				
Pre-BMT	−	−	245 (72.7)	−
Post-BMT			92 (27.3)	
Within 30 days			3 (0.89)	
30 days to 1 year			37 (11.0)	
After 1 year			52 (15.4)	
T-score at first DXA				
Spine	− 1.01 ± 1.04	− 0.381 ± 1.47	− 0.560 (− 1.40–0.480)	0.058
Femoral neck	− 1.26 ± 1.01	− 0.980 (− 1.77–0.0750)	− 1.00 (− 1.79–0.0200)	0.23
Total hip	− 0.703 ± 0.743	− 0.540 (− 1.40–0.400)	− 0.590 (− 1.39–0.390)	0.48
Donor				
Related	9 (50.0)	174 (54.5)	183 (54.3)	0.92
Unrelated	7 (38.9)	124 (38.9)	131 (38.9)	
Cord blood	1 (5.56)	15 (4.70)	16 (4.75)	
Unknown	1 (5.56)	6 (1.88)	7 (2.08)	
Total body irradiation				
Yes	6 (33.3)	105 (32.9)	111 (32.9)	1.0
No	11 (61.1)	202 (63.3)	213 (63.2)	
Unknown	1 (5.56)	12 (3.76)	13 (3.86)	
Graft versus host disease				
Yes	15 (83.3)	232 (72.7)	247 (75.5)	0.42
Gastrointestinal	1 (5.56)	39 (12.2)	40 (11.9)	
Other	14 (77.8)	193 (60.5)	207 (61.4)	
No	3 (16.7)	87 (27.3)	90 (26.7)	
Antiresorptive post BMT				
Yes	9 (50)	13 (4.08)	22 (6.53)	< 0.0001
No	9 (50)	306 (95.9)	315 (93.5)	
Timing of second DXA				
Year 0–1			216 (64.1)	
Week 0–12	−	−	10 (2.97)	−
Week 13–24			173 (51.3)	
Week 25–52			33 (9.79)	
Year 1–2			63 (18.7)	
Year 2–3			13 (3.86)	
After year 3			45 (13.4)	
Median duration of follow-up (IQR, years)	13 (10–20)	11 (8.2–14)	11 (8.2–15)	0.047
Annualised decline in areal bone mineral density (aBMD, g/cm^2^/year)^b^				
Spine	0.037 (0.0011–0.13)	0.049 (− 0.0037–0.16)	0.049 (− 0.0032–0.16)	0.93
Femoral neck	0.014 (0.0015–0.090)	0.071 (0.0043–0.17)	0.066 (0.0038–0.17)	0.10
Total hip	0.065 (0.00089–0.13)	0.095 (0.017–0.20)	0.094 (0.013–0.19)	0.17
Percentage decline in aBMD between first and second DXA				
Spine	2.6 (1.6–4.0)	3.3 (− 0.90–6.5)	3.2 (− 0.68–6.4)	0.88
Femoral neck	3.3 (0.76–6.7)	4.6 (1.1–8.3)	4.6 (1.1–8.2)	0.41
Total hip	2.6 (1.8–5.9)	4.8 (2.1–8.2)	4.7 (2.1 − 8.2)	0.20
Absolute decline in Z-score between first and second DXA				
Spine	0.17 (− 0.068–0.48)	0.28 (− 0.10–0.60)	0.27 (− 0.10–0.59)	0.62
Femoral neck	0.19 (− 0.075–0.39)	0.30 (0.035–0.62)	0.30 (0.030–0.60)	0.17
Total hip	0.19 (0.0075–0.36)	0.34 (0.12– 0.61)	0.32 (0.12–0.60)	0.081

Data are presented as n (%), mean ± SD for normally distributed variables and median (IQR) for non-normally distributed variables

^a^Missing data: weight for 48 individuals, height for 45 individuals, BMI for 83 individuals

^b^Annualised decline calculated using the difference in aBMD between the first and second DXA, proportioned to one year. Significant difference between the sites with *p* < 0.0001 as calculated using Friedman’s test

Vitamin D data were available for 244 of 337 (72%) patients. Among the 119 patients not on vitamin D supplementation, the mean (SD) 25-hydroxyvitamin D (25-OHD) level was 81nmol/L (26). Among the 125 patients on vitamin D supplementation, the median (IQR) 25-OHD level was 68nmol/L (55–87).

Admission data were available for 259 of 337 (77%) patients. The median (IQR) number of admissions was two (1–4) in the year following BMT, including the index admission for BMT. The median (IQR) number of days admitted to hospital in the year following BMT was 29 days (22–46). Of the 259 patients with admissions data, 193 had their second DXA performed in the first year post BMT. There was no correlation between days spent admitted to hospital in the first year post BMT and the annualised decline in aBMD at any site (no statistically-significant correlation using Spearman’s test).

**Fig. 1:**
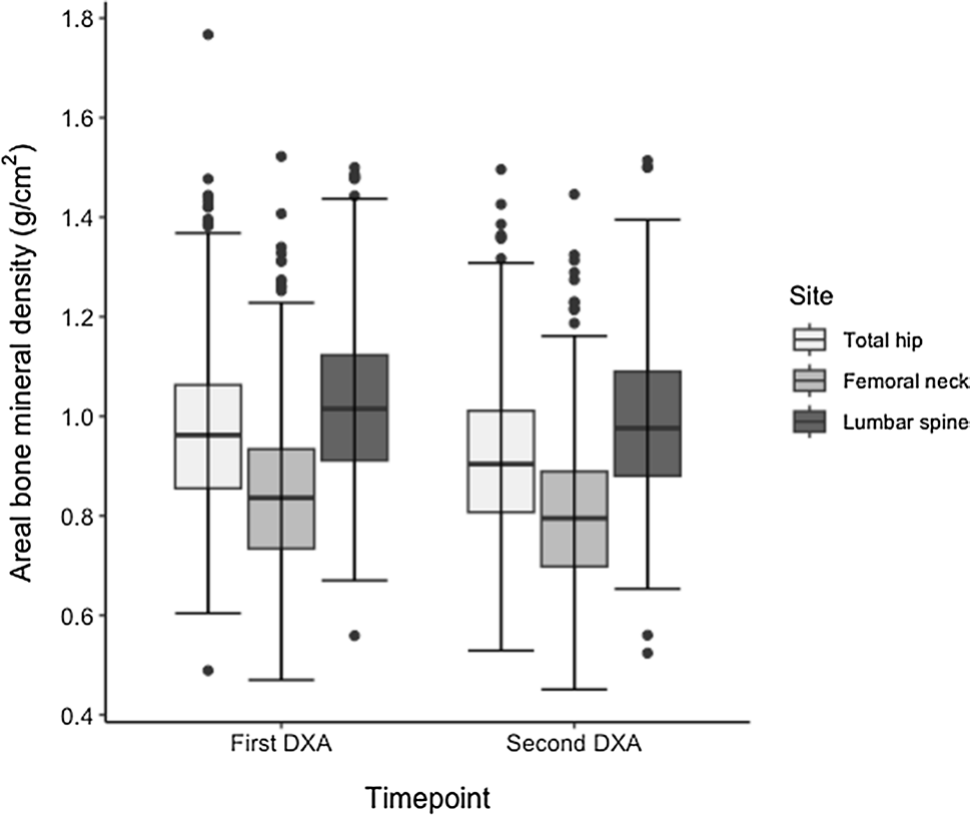
Differences in areal bone mineral density (aBMD, g/cm^2^) at the time of first and second DXA scan in 337 patients at the total hip, femoral neck and lumbar spine. Boxplots depict the median and interquartile range, with outliers depicted as dots

First available DXA T-scores for aBMD were relatively preserved: median (IQR) T-score − 0.56 (− 1.40–0.48) at the lumbar spine, − 1.00 (− 1.79–0.02) at the femoral neck, and − 0.59 (− 1.39–0.39) at the total hip. Most patients had their second DXA scan performed in the first year post BMT (216/337, 64.1%). Comparing aBMD between the first and second DXA scans, there was a significant decline in bone density at all sites (Fig. 1). The percentage decline in aBMD between first and second DXA, and the median (IQR) annualised decline in aBMD was greater at the femoral neck (4.6%; 0.066g/cm^2^ (0.0038–0.17)) and total hip (4.7%; 0.094g/cm^2^ (0.013–0.19)), compared to the spine (3.2%; 0.049g/cm^2^ (− 0.0032–0.16)). There was greater annualised decline in aBMD within the first year post BMT comparative to follow-up DXA scans performed later in the follow-up period (see Table 2, *p* < 0.001 at all sites).

**Table 2:** Annualised decline in aBMD in patients who were less than or equal to, or greater than 12 months post BMT at the time of the second DXA scan

	Less than or equal to 12 months since BMT at second DXA scan (*N* = 216)	Greater than 12 months post BMT at second DXA scan (*N* = 121)	Total (*N* = 337)	*p*
Annualised decline in aBMD (g/cm^2^/year)				
Spine	0.11 (0.00–0.21)	0.0048 (− 0.0045–0.047)	0.049 (− 0.0032–0.16)	< 0.001
Femoral neck	0.12 (0.036–0.22)	0.0094 (0.00–0.059)	0.066 (0.0038–0.17)	< 0.001
Total hip	0.14 (0.078–0.25)	0.013 (0.00–0.066)	0.094 (0.013–0.19)	< 0.001

Data expressed to 2 significant figures

**Fig. 2:**
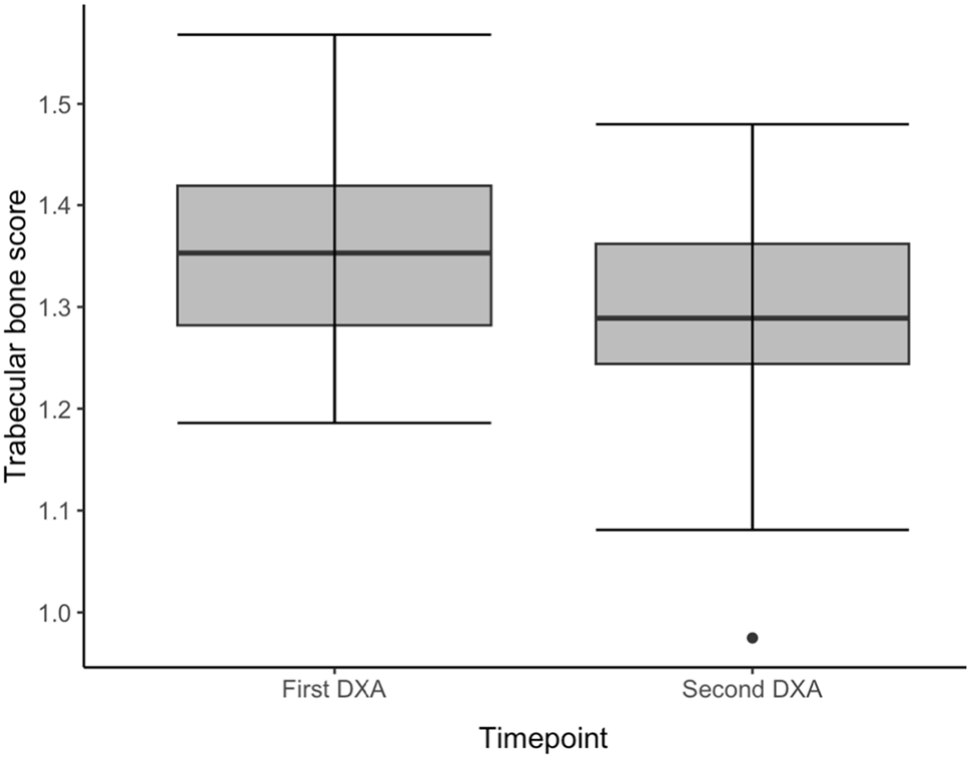
TBS at the time of first and second DXA scan in 47 patients with sequential TBS data available. Boxplots depict the median and interquartile range, with outliers depicted as dots

TBS declined significantly post BMT (Fig. 2) and independently of T-scores (no statistically-significant correlation using Spearman’s test). At baseline pre-BMT, mean TBS was 1.35 ± 0.10, while at the time of first follow-up DXA, mean TBS was 1.29 ± 0.11 (*p* = 0.006). While there is no established reference range for men, for postmenopausal women this would translate to a transition from normal to partially degraded microarchitecture following BMT.

**Fig. 3:**
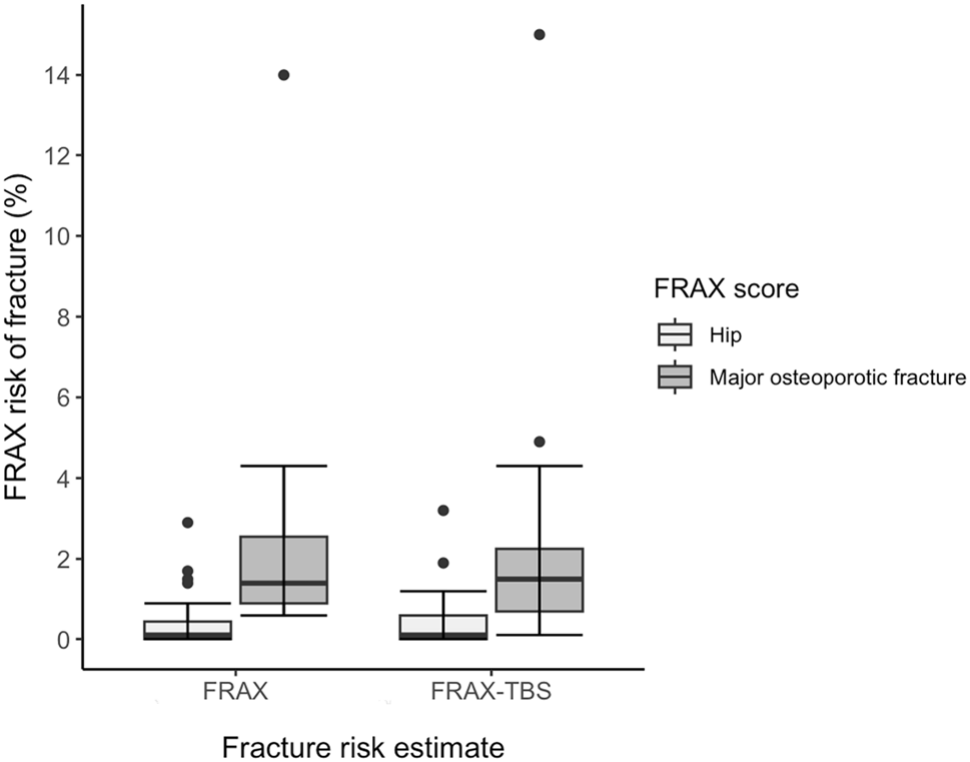
FRAX® 10-year probability of fracture (%) with and without adjustment for TBS for major osteoporotic fracture and hip fracture (*N* = 47). Despite FRAX being validated for age above 40 years, the *N* of 47 includes two people aged 37 and 38 years-old who were included given they were close to 40 years of age

Eighteen patients (5.3%) sustained 19 fractures over 3884 person-years of follow-up post-transplant. These fractures occurred at the radius/ulna (8/19), spine (3/19), femur/hip (2/19), tibia (2/19), rib (2/19) and shoulder (2/19). The median (IQR) time to fracture post BMT was 7.4 years (2.9–12). This corresponds to a 5.3% prevalence of fractures over a median follow-up of 11 years and was significantly greater than predicted by the FRAX® fracture probability calculator even after adjustment for TBS, although glucocorticoid exposure data were limited and designated “no” if unknown. When FRAX® was adjusted for TBS, there was a non-significant increase in the 10-year estimated probability of fracture from 2.69% to 2.74% (*p* = 0.53) for major osteoporotic fracture, and 0.72% to 0.83% (*p* = 0.96) for hip fracture (Fig. 3). Females were significantly more likely to fracture than males (*p* = 0.036), and those who fractured were significantly older (*p* = 0.024). There was no difference in annualised aBMD decline between fracturing and non-fracturing groups on univariate (Table 1) or multivariate (Tables 3, 4 and 5) analyses. This lack of difference persisted even after narrowing the data to patients who had their second DXA scan in the first year post BMT only (Table 4), and when patients who received antiresorptive therapy were excluded (Table 5). There was no difference in annualised TBS decline between fracturing and non-fracturing groups.

**Table 3:** Logistic regression model with age, sex and annualised aBMD decline as the independent variables used to predict probability of osteoporotic fracture post BMT

	Model 1 (Spine aBMD)		Model 2 (Femoral neck aBMD)		Model 3 (Total hip aBMD)	
	Coefficient	*p*	Coefficient	*p*	Coefficient	*p*
Gender (female)	− 1.2	0.018	− 1.2	0.022	− 1.2	0.022
Age	0.046	0.039	0.045	0.046	0.045	0.045
Annualised decline in aBMD	− 0.98	0.60	− 3.2	0.17	− 1.4	0.51

Data are expressed to 2 significant figures

**Table 4:** Logistic regression model with age, sex and annualised aBMD decline as the independent variables used to predict probability of osteoporotic fracture post BMT, for patients who had their second DXA scan completed in the first year post BMT

	Model 1 (Spine aBMD)		Model 2 (Femoral neck aBMD)		Model 3 (Total hip aBMD)	
	Coefficient	*p*	Coefficient	*p*	Coefficient	*p*
Gender (female)	− 0.44	0.50	− 0.47	0.47	− 0.44	0.50
Age	0.035	0.22	0.032	0.26	0.034	0.23
Annualised decline in aBMD	0.017	0.99	− 2.2	0.37	− 0.090	0.97

Data are expressed to 2 significant figures

**Table 5:** Logistic regression model with sex and annualised aBMD decline as the independent variables used to predict probability of osteoporotic fracture post BMT in patients who did not receive antiresorptive therapy

	Model 1 (Spine aBMD)		Model 2 (Femoral neck aBMD)		Model 3 (Total hip aBMD)	
	Coefficient	*p*	Coefficient	*p*	Coefficient	*p*
Gender (female)	− 1.2	0.10	− 1.1	0.12	− 1.1	0.12
Age	0.034	0.24	0.033	0.26	0.032	0.27
Annualised decline in aBMD	− 0.60	0.80	− 3.5	0.28	− 2.7	0.40

Data are expressed to 2 significant figures

At the time of the second DXA scan (*N* = 337), 72 patients (21%) had osteoporosis and 156 (46%) had osteopenia. Only 109 patients (22%) had normal bone density at all sites. Twenty-two (6.5%) received antiresorptive therapy. Of the 18 patients who fractured, nine received antiresorptive treatment (mean T-score − 1.9 at the lumbar spine, − 2.0 at the femoral neck and − 1.2 at the total hip at the time of the second available DXA). Thirteen of the 319 patients who did not fracture also received antiresorptive therapy (mean T-score − 2.0 at the lumbar spine, median T-score − 2.2 at the femoral neck and − 2.0 at the total hip).

## Discussion

To our knowledge, this is the first study to illustrate the bone loss, microarchitectural decay and increased fracture risk in BMT recipients over an extended follow-up period. In this group, there was rapid, significant bone loss post BMT, most pronounced in the first year following transplant, mirroring the timeline of glucocorticoid-induced osteoporosis [8]. Notably however, bone loss was greatest at the femoral neck, while glucocorticoid-induced osteoporosis predominantly affects the spine [9]. This is consistent with previous studies [3]. Decay in bone microarchitecture was also noted as evidenced by the decline in TBS post BMT. TBS decline was independent of T-score decline, as has previously been demonstrated in renal transplant recipients [10]; TBS and aBMD are therefore complementary methods for assessing bone quality.

Similar to previous studies of BMT recipients [11, 12], the femoral neck and hip sites had greater annualised bone loss than the lumbar spine. This may be partially explained by immobility-related bone loss, where demineralisation of the weight-bearing skeleton including the femur and tibia lead to reduced bone density and increased fracture risk at these sites [13, 14]. Exercise has been shown to improve function, quality of life and length of stay in BMT recipients [15]. In our study, BMT recipients were admitted for a median of one month in the year after transplant, however frequency of admission did not correlate with annualised decline in aBMD. Research into other pathophysiological mechanisms is warranted, including investigation of vitamin D status post BMT. Some studies have suggested vitamin D deficiency may be prevalent in BMT recipients [16], although this was not the case in our cohort.

This deterioration in bone health translates to an increased fracture risk. Of concern, these patients are fracturing despite relatively preserved aBMD and TBS at the time of first DXA scan. We recorded 19 fractures over 3884 person years of follow-up, which equates to a 5.3% probability of osteoporotic fracture over the median 11-year study period. Some studies have reported an even greater risk, with up to 8–10.6% of BMT recipients experiencing fragility fractures [17, 18]. This high probability of fracture in the BMT subpopulation is greater than that estimated by conventional fracture probability calculators including FRAX®, which do not include BMT as a risk factor. Glucocorticoids have been shown to increase fracture risk independent of aBMD [19], and similarly our study found fracture incidence was independent of annualised aBMD decline.

TBS is a novel and useful adjunct that can assist clinicians in predicting future fractures. FRAX®-TBS predicted a higher (although not significant) probability of fracture compared to FRAX® alone. TBS is known to improve fracture risk assessment in glucocorticoid-induced osteoporosis and, given that this is a factor in BMT recipients, further supports TBS utility in the BMT population [20].

There are few studies of TBS in BMT patients and they have contrasting findings. Pawlowska et al. [21] evaluated 137 patients to find that TBS declined significantly in women and patients treated with glucocorticoids. Conversely in 68 patients, Lim et al. [3] found no significant change in TBS from baseline to 12 months and from 12 to 24 months, although the sample size was small. Further study of TBS in the BMT population could assess its utility in guiding earlier appropriate antiresorptive treatment. Currently, there are no accepted thresholds for TBS alone to prompt antiresorptive therapy, although this may change in the future. Instead, its present utility is in augmenting fracture probability estimates, e.g. FRAX®-TBS, or qualitatively when considered in conjunction with aBMD.

Our study is limited by being a retrospective, single-centre cohort study. There were also limited glucocorticoid exposure data available, which may have contributed to an underestimate of FRAX fracture risk. The relatively younger age of individuals post BMT may have also contributed to an underestimated fracture risk, with younger BMT recipients less likely to fracture than older recipients [18]. The study is strengthened by a relatively large sample size with substantive longitudinal follow-up and the ability to assess fracture occurrence. While there were only 47 patients with sequential TBS available in our study, there are limited data on TBS in the BMT population and thus this represents a relatively sizeable population. There were a small number of patients who fractured, which may limit comparisons between the group of patients who did and did not fracture. For patients who did not respond to the survey, fracture data were collected from the medical record and this method may have missed some fractures that occurred in the community. However, this study provides a fresh perspective on fracture probability and TBS utility in this population that may be confirmed with future research endeavours.

Patients post BMT are vulnerable and experience long-term health consequences post-transplant. This includes a high risk of declining bone health and fracture, which is multifactorial. Recognising declining bone health early is paramount as it allows earlier institution of effective and well-tolerated antiresorptive therapies where appropriate. The FRAX algorithm was derived from patients older than forty years of age, and is therefore less applicable to BMT patients who are younger than other solid organ transplantation cohorts such as kidney transplant recipients [22].

Unlike solid organ transplant recipients who are often on lifelong glucocorticoids, BMT subjects are weaned off immunosuppressants. Therefore, fracture calculators underestimate absolute fracture risk because they do not register BMT recipients with additional risk factors either via prolonged high-dose glucocorticoids use or the presence of secondary osteoporosis with chronic conditions such as type 1 diabetes or chronic liver disease. An additional adjustment factoring BMT into fracture probability calculators may improve bone health assessment in the future. We propose a factor adjustment by at least 100–200% based on the 5.3% fracture prevalence in our study compared to the 2.69% FRAX estimate although this may be an underestimate due to limited glucocorticoid data. This may encourage earlier intervention with antiresorptive therapy where appropriate, thereby minimising preventable fractures.

The current recommendation post BMT is to assess aBMD using DXA at baseline and within one year post-transplant or at 3 months if there has been exposure to high-dose corticosteroids early post-transplant [6, 23]. Newer guidelines recommend antiresorptive therapy if DXA T-score < 1.5 [24]. The guidelines in solid organ recipients recommend antiresorptive therapy in those with osteopenic T-score to offset the rapid bone loss immediately post-transplantation. Our study demonstrated rapid aBMD decline in the first year post BMT independent of TBS decline, therefore we recommend a DXA scan with TBS at baseline and within one year post BMT with subsequent DXA scans guided by the clinical picture.

Akin to the current literature, prophylactic antiresorptive therapy was not administered to many patients with an indication for treatment. Eighty-four patients had clinically-defined osteoporosis, either having T-scores in the osteoporotic range or a low-trauma fracture plus osteopenic aBMD, providing an indication for antiresorptive therapy. Seventeen of these patients received antiresorptive, corresponding with a 20% treatment rate. In the BMT population, multidisciplinary collaboration and further research may improve fracture risk calculation and earlier antiresorptive therapy where appropriate.

## Conclusion

In BMT patients, aBMD and TBS declined rapidly and independently during the first year post BMT; however, FRAX® fracture probability estimates incorporating these values significantly underestimate fracture rates and osteoporosis treatment rates remain low. It is recommended that patients undergo baseline DXA and follow-up DXA within 1-year post BMT, preferably incorporating TBS, to risk stratify for osteoporosis therapy. FRAX® fracture probability calculations should be considered underestimates. In the future, specific adjustments for BMT patients may enhance predictions of fracture risk and facilitate optimal use of osteoporosis therapies.

### Supplementary Information

Below is the link to the electronic supplementary material.

Supplementary material 1 (DOCX 60.3 kb)

## Abbreviations

aBMD: Areal bone mineral density
BMI: Body mass index
BMT: Bone marrow transplant
DXA: Dual energy X-ray absorptiometry
GVHD: Graft-versus-host disease
25-OHD: 25-hydroxyvitamin D
IQR: Interquartile range
RMH: The Royal Melbourne Hospital
SD: Standard deviation
TBS: Trabecular bone score
