# Supplementary material for: Bone Density and Trabecular Bone Score Decline Rapidly in the First Year After Bone Marrow Transplantation with a Marked Increase in 10-Year Fracture Risk
Source: Calcif Tissue Int. 2024 Feb 20;114(4):377–85. doi: 10.1007/s00223-024-01189-1 (PMC10957585; doi:10.1007/s00223-024-01189-1)
Supplement: Supplementary file 1 — Supplementary material 1 (DOCX 60.3 kb) [file 223_2024_1189_MOESM1_ESM.docx]

**Supplementary Appendix 1 – Patient survey**

**This section is about work**

1. What is your current occupation?

|  |
| --- |

**The next section asks for details about your fractures.**

1. How many fractures have you had?

*If you broke multiple bones at the same time e.g., you were in a motor vehicle accident and broke your arm and your leg, please consider these to be a single fracture event and enter these in together. We especially want to know whether you have had any fractures involving the spine (called vertebral compression fractures), wrist/forearm, hip, upper arm, lower leg, and pelvic bones.*

If none, skip to **page 5, Q10**

1. Which bone(s) did you fracture?

|  |
| --- |

1. Approximately when did this fracture occur?

*If you can't remember the exact date that's ok, just enter the month and/or year.*

|  |
| --- |

1. How did this/these fracture/s occur?

*We want to know whether you had what are called a low trauma or a high trauma fracture. For example, falling down a flight of stairs is a high trauma situation but tripping over the pavement and falling from a standing height or lower is a low trauma situation. As much detail as you can provide here is beneficial.*

|  |
| --- |

1. How did your doctors confirm you had this/these fracture(s)?

*To the best of your knowledge, we want to know how your doctors confirmed your fracture(s). Was it through an X-ray scan (also called plain film), CT scan (also called CAT scan), or an MRI scan? If you don’t know, that’s perfectly fine.*

|  |
| --- |

1. Were you admitted to the hospital overnight for this/these fracture(s)?

Yes  No

1. If you were admitted to the hospital, approximately how many nights did you stay?

*Please include time spent in rehabilitation and please specify for each fracture.*

|  |
| --- |

1. Did you have any surgeries to treat this fracture?

*Please specify for each fracture.*

|  |
| --- |

**This next section is about your osteoporosis status**

1. Have you ever been diagnosed by a medical professional with osteoporosis (weak bones)?

Yes  No

If *no*, skip to **page 6, Q13**.

1. Have you ever received any of the following treatments for osteoporosis?

***Alendronate (Alendrobell, Densate, Dronalen, Fonat, Fosamax)*** - Daily or weekly tablet, may be in a combination pack with vitamin D +/- calcium tablets

***Risedronate (Acris, Actonel, Atelvia, Risedro)*** - Daily, weekly, or monthly tablet, may be in a combination pack with vitamin D

***Denosumab (Prolia)*** - 6-monthly injection into the skin of the abdomen

***Zoledronic Acid (Aclasta, Zometa)*** - Yearly intravenous infusion through a drip

***Teriparatide (Forteo)*** - Daily injection into the skin of the abdomen

***Raloxifene (Evifyne, Evista, Fixta)*** - Daily tablet, usually only prescribed to females

***Strontium ranelate (Protos)*** - Daily sachet, dissolved in water

None of the above

1. For how long did you receive your medication(s)?

|  |
| --- |

**The following questions will ask about your medical history**

1. Listed below are some medical conditions that may impact bone health. Please tick the box if any of the below options apply to you (please tick all options that apply).

Parent who has had a hip fracture previously

Current or previous regular cigarette smoker

Alcohol intake >2 standard drinks a day (use this link if needed: adf.org.au/insights/what-is-a-standard-drink/)

Two or more falls in the past 6 months

Two or more falls in the past 12 months

History of requiring food via an IV drip or a feeding tube

History of being underweight/low body weight (BMI <20) (use this link if needed: [www.bupa.com.au/healthlink/health-tools/bmi-calculator](http://www.bupa.com.au/healthlink/health-tools/bmi-calculator))

Menopause before 40 years of age

Hypogonadism (low testosterone levels)

Rheumatoid arthritis

Hyperthyroidism (high thyroid hormone levels, e.g. Grave's disease)

Hyperparathyroidism (high blood calcium levels)

Chronic kidney disease (kidney failure)

Chronic liver disease (cirrhosis)

Coeliac disease or malabsorption

Diabetes (including diabetes due to steroid use)

HIV infection

1. Listed below are some treatments that may impact bone health. Please tick the box if any of the below options apply to you (please tick all options that apply).

***Aromatase inhibitors (medications used to treat breast cancer)*** - e.g. anastrozole (Anastrol) , exemestane (Aromasin), letrozole (Gynotril)

***Hormone replacement therapy or menopausal treatment therapy*** - e.g. oestrogen tablets or patches (excluding the oral contraceptive pill/OCP)

***Anti-androgen therapy (medication that decreases testosterone levels and is used to treat prostate cancer)*** - e.g. goserelin (Zoladex), degarelix (Firmagon), enzalutamide (Xtandi)

***Anti-epileptic medication (medications used to treat epilepsy)*** - e.g. levetiracetam (Keppra), carbmazepine (Tegretol), valproate (Epilim), phenytoin (Dilantin)

***Anti-depressant medication (medications used to treat depression/anxiety)*** - e.g. sertraline (Zoloft), fluoxetine (Lovan), escitalopram (Lexapro)

***Anti-psychotic medication (medications used to treat mental health conditions)*** - e.g. quetiapine (Seroquel), olanzapine (Zyprexa), risperidone (Risperdal)

***Proton-pump inhibitors (medications used to treat reflux)*** - e.g. pantoprazole (Somac), esomeprazole (Nexium)

***Thyroid hormone replacement*** - e.g. thyroxine/levothyroxine (Oroxine, Eutroxsig)

***Calcium supplements*** - e.g. Caltrate

***Vitamin D supplements*** - e.g. Caltrate Plus, Ostelin + Calcium

1. If you selected any of the previous options, please provide any additional information below.

*For example - when you were diagnosed with this condition, the name of the medication your are taking within the above category, or how many cigarettes you have smoked per day for how many years. Don't worry if you can't remember much about these conditions, any information you can remember is useful.*

|  |
| --- |

**This next section will ask about your calcium intake**

*The following image shows examples of dietary sources of calcium, most of which are dairy-based foods, with some dairy alternatives beneath them.*


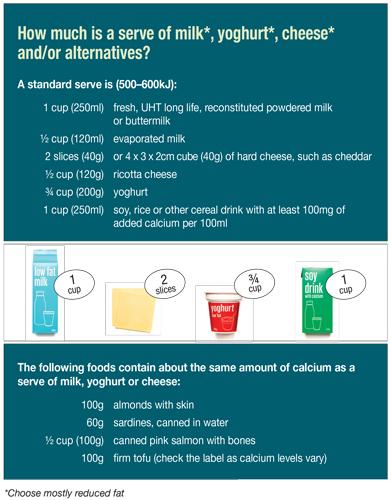

*From:* [*https://www.eatforhealth.gov.au/food-essentials/five-food-groups/milk-yoghurt-cheese-andor-their-alternatives-mostly-reduced-fat*](https://www.eatforhealth.gov.au/food-essentials/five-food-groups/milk-yoghurt-cheese-andor-their-alternatives-mostly-reduced-fat)

1. How many times a day would you consume a "serve" of dairy (or other alternative)? Please use the previous image as a guide to help you answer this question if needed.

Once a day

Twice a day

Three or more times a day

**The next section will be about your physical activity**

1. How many times a week do you exercise?

I do not exercise regularly

Once a week

Twice a week

Three times a week

Four times a week

Five times a week

Six times a week

Every day/Seven times a week

1. How much time do you spend exercising?

I do not exercise regularly

15 minutes

30 minutes

45 minutes

1 hour or longer

1. Regarding the type of physical activity, on average, how intense is the exercise that you are completing?

I do not exercise regularly

***Low intensity*** - can speak in full sentences during exercise, do not get "puffed"

***Medium intensity*** - can speak in sentences during exercise but is difficult and you cannot sustain this, get a "bit puffed"

***High intensity*** - unable to speak in sentences, out of breath, are "puffed"
